# Establishing trimester-specific reference intervals for coagulation parameters in pregnant women in China

**DOI:** 10.1186/s12884-026-09296-7

**Published:** 2026-05-18

**Authors:** Long Zhang, Yifang Shen, Kaiqi Wu, Wei Wang, Xingjun Meng, Bo Zhu

**Affiliations:** Department of Clinical Laboratory, Women’s Hospital, School of Medicine, Zhejiang University, 1 Xueshi Road, Hangzhou, 310006 China

**Keywords:** D-dimer, Plasminogen, Thrombin-antithrombin complex, Pregnancy, Reference interval

## Abstract

**Background:**

During pregnancy, the body’s hemorrhagic and coagulation system undergoes significant changes. Reference intervals for coagulation parameters may vary across different study populations and with different testing instruments. This study aimed to establish reference intervals for coagulation parameters in non-pregnant and pregnant women in China.

**Methods:**

Between January 2023 and February 2024, blood samples from 879 pregnant women and 253 healthy non-pregnant women were tested. The parameters tested included activated partial thromboplastin time (APTT), prothrombin time (PT), thrombin time (TT), fibrinogen (FBG), D-dimer, fibrin/fibrinogen degradation products (FDP), Plasminogen (PLG), thrombin-antithrombin complex (TAT), thrombomodulin (TM), plasmin-α2 plasmin inhibitor complex (PIC), and tissue plasminogen activator/plasminogen activator inhibitor-I complex (t-PAIC). Reference intervals were established using a non-parametric method.

**Results:**

The values of PT, INR, APTT, and TT decreased, while the concentrations of fibrinogen, D-dimer, FDP, PLG, TM, TAT, PIC, and t-PAIC increased significantly with advancing gestational age. Distinct reference intervals were established for healthy non-pregnant women and for women in the first, second, and third trimesters of pregnancy. Most intervals differed significantly across these groups.

**Conclusions:**

Reference intervals for non-pregnant women and pregnant women in different trimesters vary. Description of the reference intervals according to trimester of pregnant women in China will help clinicians recognise abnormality and inform medical decision-making.

**Supplementary Information:**

The online version contains supplementary material available at 10.1186/s12884-026-09296-7.

## Background

Pregnancy is a complex physiological process unique to women and marked by a host of changes in the body [1, 2]. During this period, a state of hypercoagulability develops due to enhanced coagulation and relatively weakened anticoagulation, thereby increasing the risk of thrombosis [3–5]. By identifying and addressing the factors that contribute to a hypercoagulable state during pregnancy, we can help mitigate the risks and improve the health outcomes for women and their babies.

Reference intervals for traditional coagulation parameters during pregnancy, such as activated partial thromboplastin time (APTT), prothrombin time (PT), thrombin time (TT), fibrinogen (FBG), and D-dimer(DD), have been previously reported [6–8]. However, conventional tests like PT, APTT, TT, and FBG are more effective in detecting hypocoagulability than hypercoagulability, limiting their utility in managing pregnancy-associated thrombosis [9, 10]. D-dimer is commonly used as a sensitive marker in the diagnostic workup for thrombosis due to its high negative predictive value [11, 12]. Nonetheless, its levels increase physiologically with gestational age, complicating its interpretation and application in pregnancy [13–15]. Therefore, there is a clinical need for more stable, reliable, and sensitive markers to evaluate hemostatic balance and associated risks.

Novel coagulation-fibrinolysis indices—including plasminogen (PLG), thrombin-antithrombin complex (TAT), thrombomodulin (TM), plasmin-α2 plasmin inhibitor complex (PIC), and tissue plasminogen activator/plasminogen activator inhibitor-I complex (t-PAIC)—offer potential in addressing this gap.

**Fig. 1:**
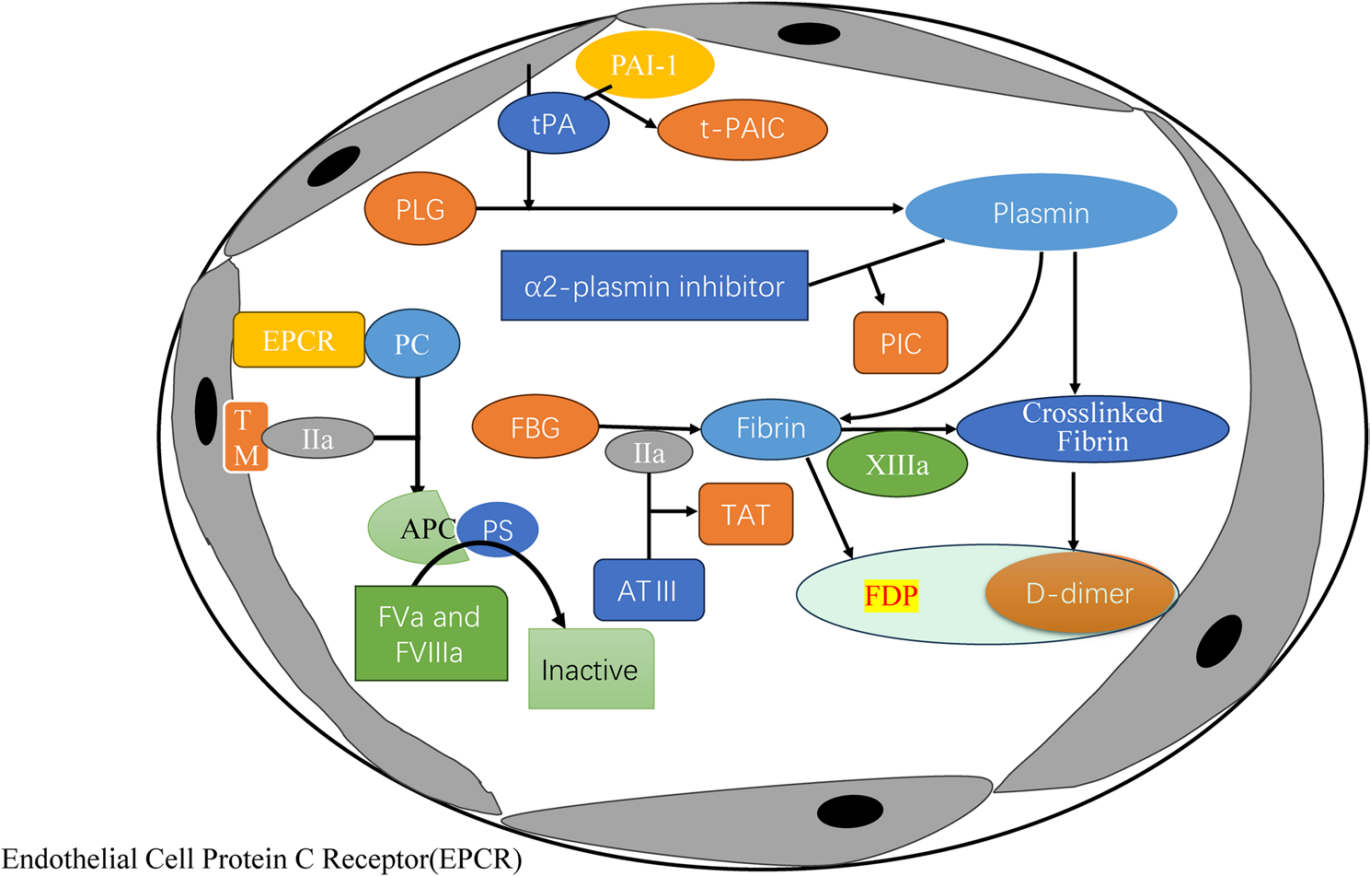
Schema of coagulation reactions. (The process of blood clot formation and degradation involves several steps. It begins with the activation of fibrinogen by thrombin (IIa), resulting in the formation of a fibrin monomer. This monomer, aided by fibrin-stabilizing factors (XIIIa), creates cross-linked fibrin. Plasminogen (PLG) is activated by tissue plasminogen activator (t-PA) to form plasmin, which breaks down fibrinogen and cross-linked fibrin to produce fibrin degradation products (FDP). The degradation of cross-linked fibrin by plasmin produces a substance called D-dimer. However, t-PA can be inhibited by plasminogen activator inhibitor-1 (PAI-1) to form a t-PA inhibitor complex (t-PAIC). Additionally, antithrombin inactivates thrombin in a 1:1 ratio to form thrombin-antithrombin complex (TAT), while an α2-plasmin inhibitor inactivates plasmin to form plasmin-α2-plasmin inhibitor complex (PIC). Thrombomodulin (TM) prevents blood clots by deactivating proteins Va and VIIIa through a protein C-based system.)

Thrombin activates fibrinogen in the downstream pathway, but within one minute, it is inactivated by antithrombin at a 1:1 ratio to form TAT. Plasmin becomes activated to degrade fibrinogen and cross-linked fibrin, forming fibrin/fibrinogen degradation products (FDP). Plasminogen is inactivated by α2 plasmin inhibitor within a few seconds to form PIC [16, 17]. If the levels of TAT increase in the blood, it indicates that the coagulation system is activated, which increases the risk of thrombosis. Conversely, elevated PIC levels indicate activation of the fibrinolytic system, which may potentially lead to an increased bleeding risk. When fibrinogen is cleaved by thrombin, it produces a fibrin monomer that forms cross-linked fibrin with the help of fibrin-stabilizing factors. D-dimer is a by-product of the degradation of cross-linked fibrin by plasmin. The rise in D-dimer levels can be primarily attributed to the activation of either the coagulation or fibrinolytic systems. However, the predominant activation of the coagulation or fibrinolytic system indicates different stages of disease progression in the body. Thrombomodulin (TM) is a type I transmembrane protein expressed predominantly on endothelial cells. Plasma TM is secreted by endothelial cells, and elevated TM levels indicate endothelial cell damage [18]. When plasminogen activator inhibitor (PAI) inhibits tissue plasminogen activator (t-PA), a complex called t-PAIC is formed. Both PAI and t-PA are secreted by endothelial cells. Elevated levels of t-PAIC indicate damage to the endothelial cells and an inhibitory condition of the fibrinolytic system (Fig. 1).

The reference interval (RI) is a vital tool in laboratory medicine, guiding clinical decision-making. However, reported reference intervals for PLG, TAT, TM, PIC, and t-PAIC vary considerably across studies and regions. For example, while one study in Changsha, China, found no significant difference in TAT between the first and second trimesters [16], studies in Beijing and Shenzhen reported significant differences [17, 18]. This highlights the importance of establishing population-specific reference data.

Consequently, there is an urgent need to establish reliable reference intervals for these parameters in the local population. This study was conducted to establish reference intervals for both traditional and novel coagulation-fibrinolysis parameters in healthy non-pregnant and pregnant women in Zhejiang Province of China, following standardized methodology and subject selection.

## Subjects and methods

### Subjects

**Fig. 2:**
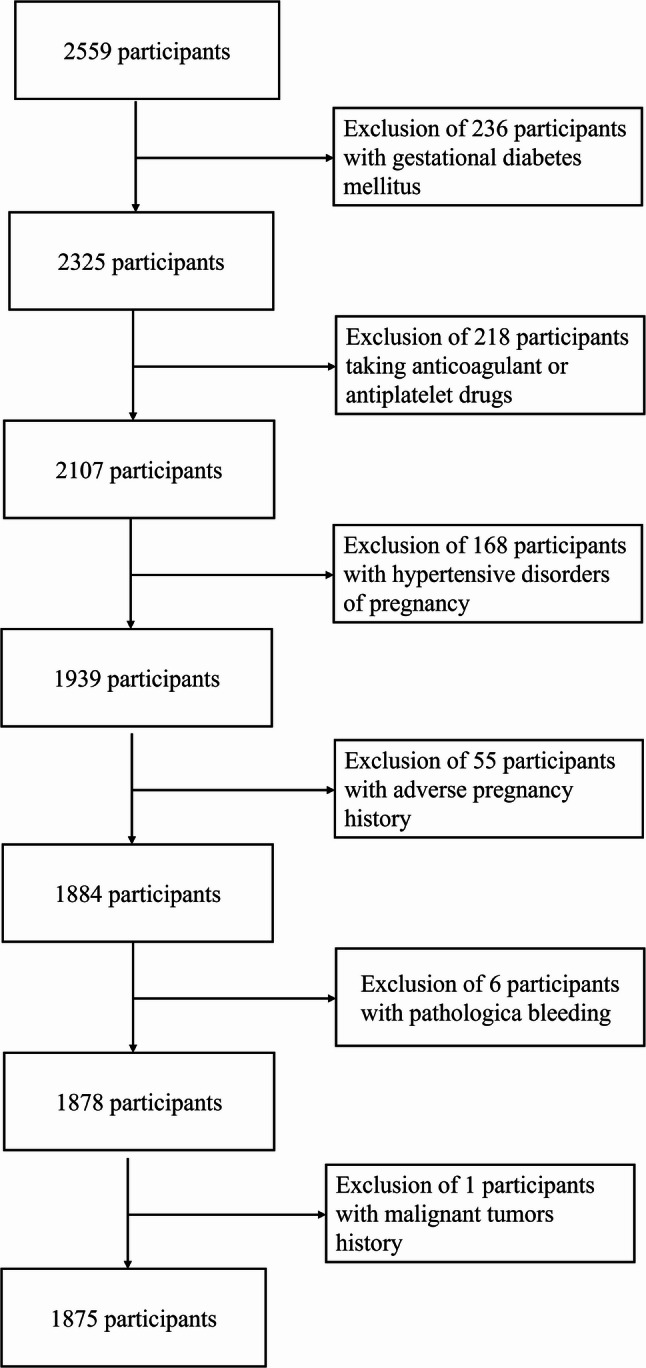
Flow chart of participant screening and inclusion. This flowchart illustrates the stepwise process of participant recruitment, screening, and final group allocation for the study. Initially, 2,559 women were recruited from the outpatient department. After applying the pre-defined inclusion and exclusion criteria, 1,875 eligible women were enrolled. The enrolled participants were then stratified into the following groups for analysis: the healthy pregnant group (*n* = 1,622), further subdivided into the first (*n *=…), second (*n *=…), and third trimester (*n* =…) subgroups based on gestational age, and the healthy non-pregnant control group (*n* = 253). The primary reasons for exclusion are listed, which included factors such as not meeting the age criteria, presence of conditions affecting coagulation parameters (e.g., specific diseases, recent infections/surgery, medication use), and other protocol-specific reasons

This cross-sectional study was conducted at the outpatient department of the Women’s Hospital, Zhejiang University School of Medicine, between January 2023 and February 2024. A total of 2559 women were recruited, of whom 1875 met the criteria and were enrolled. This included 1622 healthy pregnant women in the pregnancy group and 253 healthy non-pregnant women (NP) in the control group. The stepwise participant selection process is shown in Fig. 2. Gestational age was determined by the last menstrual period for women with regular menstrual cycles, and by first-trimester ultrasound (≤ 13⁺⁶ weeks) for those with irregular menstrual cycles.

#### Inclusion criteria

Age 18–48 years.

Healthy pregnant women: singleton pregnancy without major obstetric complications.

Healthy non-pregnant women: not pregnant, with regular menstrual cycles, and without known major systemic diseases.

#### Exclusion criteria

Individuals were excluded if they presented with any of the following conditions, which are known to affect coagulation or fibrinolysis parameters:

##### Major medical history and diseases

History of smoking.

Hypertension, heart disease, chronic kidney disease, liver disease, lung disease, pancreatic disease, or malignancy.

History of cardiovascular or cerebrovascular disease.

Immune disorders (e.g., rheumatoid arthritis, systemic lupus erythematosus, idiopathic thrombocytopenic purpura).

Diagnosed antiphospholipid syndrome.

Personal or family history of thromboembolic disease, or known coagulation abnormalities.

Acute or chronic hematologic diseases before or during pregnancy.

##### Recent clinical events and status

History of surgery or trauma within 30 days before blood sampling.

History of blood transfusion within six months before blood sampling.

Presence of infection with a body temperature > 37.5 °C within 30 days before blood sampling.

Experiencing a stressful condition within one day before blood sampling.

(For third-trimester participants) Being in active labor.

##### Medication history

Use of anticoagulant or antiplatelet drugs.

##### Obstetric-specific exclusion criteria

History of recurrent miscarriage.

History of abnormal fetal development.

Considering the conventional pregnancy classification criteria and changes in coagulation status during pregnancy, healthy pregnant women were divided into three groups as follows: the first trimester (< 14 weeks), the second trimester (14^+ 0^-27^+ 6^ weeks), the third trimester (28^+ 0^-40^+ 6^ weeks).

### Methods

#### Specimen collection

Blood samples were collected into anticoagulant tubes (BD Medical Systems, Franklin Lakes, NJ, USA) containing 0.109 mol/L sodium citrate, with a ratio of anticoagulant to venous blood of 1:9. The samples were mixed immediately and centrifuged at 2500 g for 15 min. All samples were centrifuged within 1 h of collection. Coagulation testing was completed within 2 h of blood collection. The remaining plasma samples after testing were stored in an ultra-low temperature freezer at − 80 °C for possible re-examination.

#### Instruments and detection

PT, APTT, TT, FBG (by the Clauss method), D-dimer, FDP, and PLG were assayed on the STA-R MAX coagulation analyzer (Diagnostica Stago, Asnières sur Seine, France). TAT, TM, PIC, and t-PAIC concentrations were assayed on the Shine i2900 automated chemiluminescence immunoassay (Wondfo Biotech Co., Ltd., Guangzhou, China). This equipment used original matching reagents and was cleaned and maintained daily, weekly, monthly, and irregularly per instructions. During specimen testing, control products were used daily for in-house quality control to ensure the precision of the test results. Each test was performed according to the standard operating procedures (SOP) of the instrument and completed on the analyzer within 2 h, and all specimens were tested as routine clinical specimens. The reference intervals provided by the manufacturer for each assay are listed in Supplementary Table 1. The intra‑ and inter‑assay coefficients of variation for coagulation and fibrinolysis parameters, as presented in Supplementary Table 2.

### Statistical analysis

For PT, INR, APTT, TT, FBG, PLG, and TM, two-sided reference intervals were determined nonparametrically as the 2.5th to 97.5th percentiles. For DD, FDP, PIC, TAT, and t-PAIC, one-sided upper reference limits were set at the 95th percentile. The exclusion of outliers was determined using Dixon’s test [19]. Bilateral reference intervals were used for PT, APTT, TT, FBG, D-dimer, FDP, PLG, TAT, TM, PIC, and t-PAIC, and the 2.5th percentile (P2.5) and 97.5th percentile (P97.5) were calculated as upper and lower limits of the reference intervals. We compared the PT, APTT, TT, FBG, D-dimer, FDP, PLG, TAT, TM, PIC, and t-PAIC among the four groups using the Kruskal-Wallis H test. The non-parametric Dunn’s test was used for multiple pairwise comparisons, with P values adjusted using the Benjamini-Hochberg method. Statistical Product and Service Solutions (SPSS) software (version 22.0; SPSS, Inc, IL, USA) and the R package (http://www.r-project.org) were utilized to analyze the data and create the figures. Spearman’s rank correlation test was used to analyze the correlations between gestational age and coagulation–fibrinolysis parameters. All tests were two-sided, and a P-value of less than 0.05 was considered statistically significant. The RI established in this experiment strictly follows the Clinical and Laboratory Standards Institute (CLSI) C28-A3 guideline [19].

## Results

### Characteristics of participants

Characteristics of participants in each group, including number, age, and gestational week, are detailed in Table 1 below.

**Table 1:** Characteristics of participants

	Number	Age (years)	Gestational Age(weeks) Median(IQR)
Healthy non-pregnant women	253	32 ± 5.8	/
First trimester	296	30 ± 5.6	6.14(5.57–7.68)
Second trimester	517	31 ± 4.2	24.14(19.43–25.43)
Third trimester	809	31 ± 4.1	34.10(32.02–36.82)

### Effect of the gestational week on coagulation and fibrinolysis parameters

**Fig. 3:**
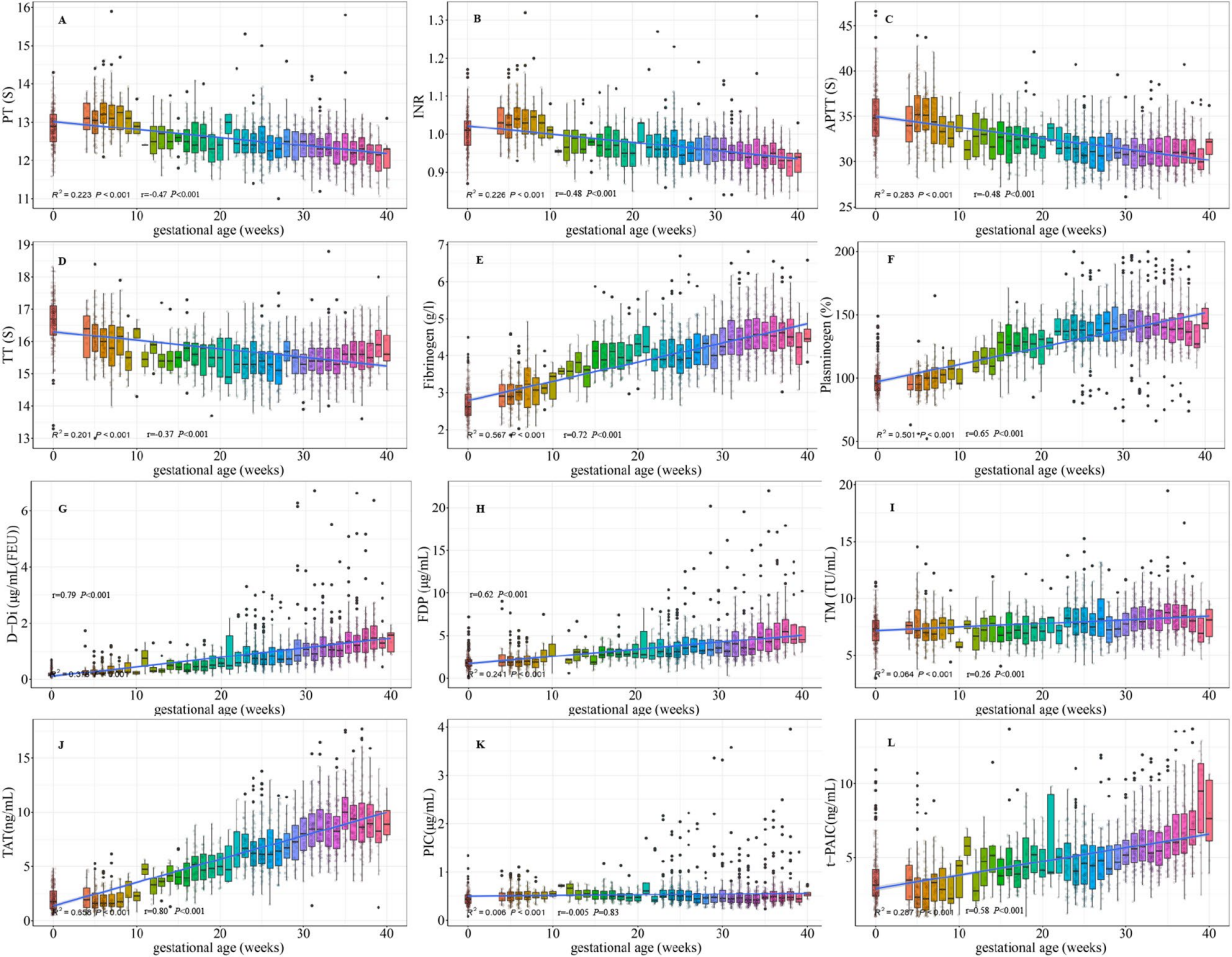
Dynamic changes in coagulation and fibrinolysis parameters during pregnancy. Each subpanel (**A**–**L**) displays the distribution of values at each gestational week using a vertical boxplot (box: interquartile range; line within the box: median; whiskers: range), overlaid with individual data points. The correlation between gestational age and coagulation and fibrinolysis parameters was analyzed using Spearman’s correlation. The blue fitted linear regression line shows the fitted curve and overall trend between gestational age and the parameters. The correlation coefficient (r), coefficient of determination (R^²^), and corresponding statistical significance (*P* value) are annotated for each parameter. All parameters showed a statistically significant trend across gestation (*P* < 0.001) except for PIC. The main patterns include a progressive decrease in PT, INR, APTT, and TT, and a progressive increase in fibrinogen, D‑dimer, FDP, TAT, PIC, and t‑PAIC

Coagulation parameters exhibited dynamic changes throughout gestation. A progressive shortening of coagulation times was observed, as indicated by the significant decrease in PT, INR, APTT, and TT values with increasing gestational week (GW), consistent with a state of advancing hypercoagulability (Fig. 3). Concurrently, the concentrations of key procoagulant and fibrinolytic factors increased. Notably, fibrinogen, plasminogen (PLG), and thrombin-antithrombin complex (TAT) levels rose significantly from as early as the 11th GW and continued to increase thereafter. Similarly, D-dimer (DD), fibrin(ogen) degradation products (FDP), and t-PAIC concentrations showed a general upward trend across pregnancy, though without distinct inflection points. In contrast, the median levels of thrombomodulin (TM) and plasmin-α2-plasmin inhibitor complex (PIC) remained relatively stable across gestation. However, greater dispersion in PIC values was noted by the 21st week, with some individuals exhibiting levels two to three times the median (Fig. 3).

### Establishment of trimester-specific reference intervals

To translate the observed physiological trends into clinically applicable tools, we established trimester-specific reference intervals for maternal coagulation parameters. The statistical approach for interval determination (as detailed in the Methods section) was applied to the stratified cohort: two-sided intervals (2.5th–97.5th percentiles) were established for parameters where deviations in either direction are clinically meaningful (PT, INR, APTT, TT, FBG, PLG, TM), while one-sided upper limits (95th percentile) were set for markers where only elevation indicates pathology (DD, FDP, PIC, TAT, t‑PAIC).

**Fig. 4:**
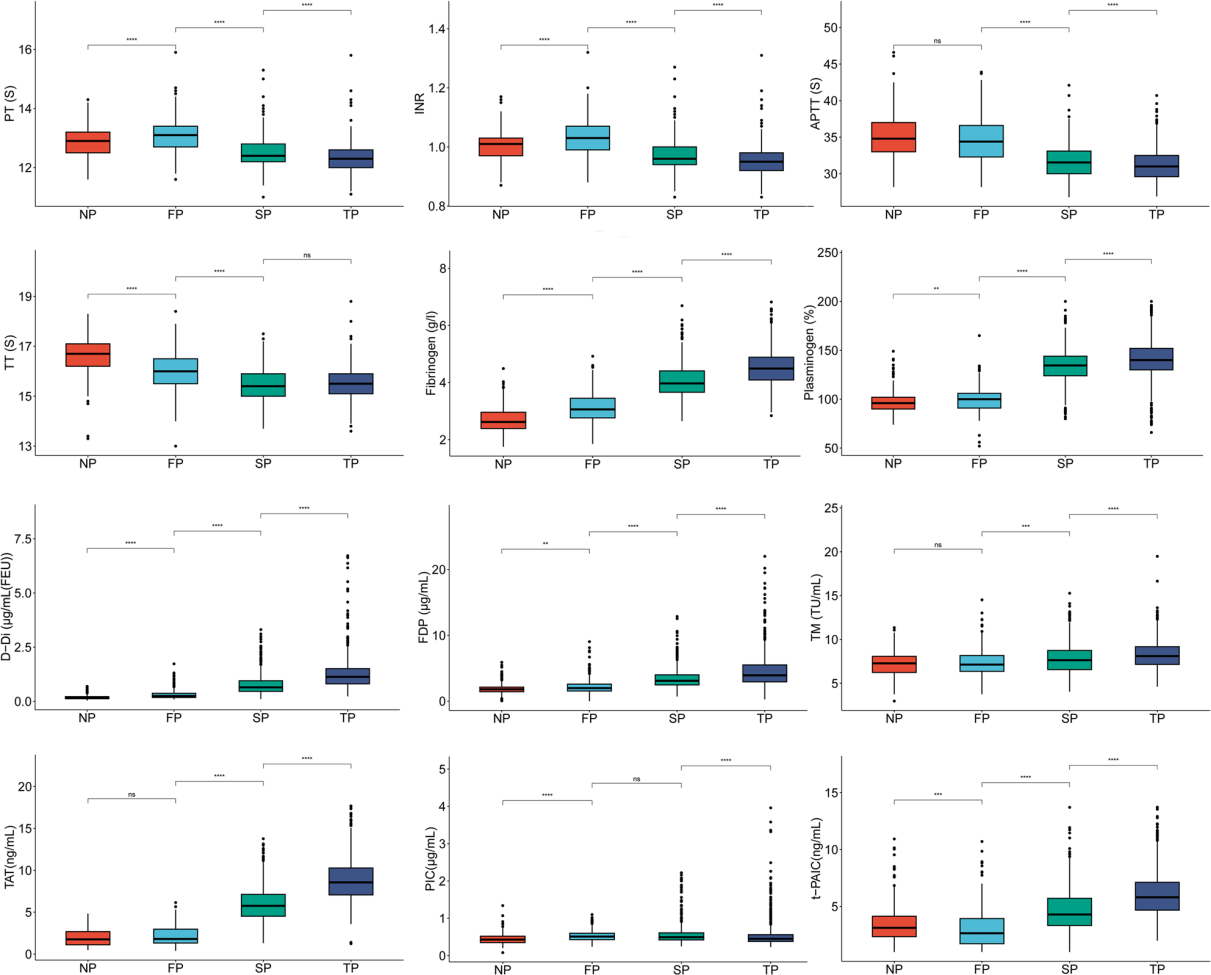
Comparison of coagulation and fibrinolysis parameter levels across pregnancy trimesters and non-pregnant controls. This figure illustrates the distributions and comparisons of 12 coagulation and fibrinolysis parameters—PT, INR, APTT, TT, FBG, D-dimer, FDP, PLG, TAT, TM, PIC, and t-PAIC—across four groups: healthy non-pregnant women (NP), first-trimester pregnant women (FP), second-trimester pregnant women (SP), and third-trimester pregnant women (TP).Statistical comparisons of median values between each pair of adjacent groups (e.g., NP vs. FP) were performed using the non-parametric Dunn’s test for multiple pairwise comparisons, with *P* values adjusted via the Benjamini-Hochberg method.Significance levels are denoted as follows: “ns” (*P* ≥ 0.05), “*” (*P* < 0.05), “**” (*P* < 0.01), and “***” (*P* < 0.001)

The resulting reference intervals are presented in Table 2. They quantitatively confirm the progressive hypercoagulable state across gestation: coagulation times (PT, INR, APTT, TT) shortened from the first to the third trimester, whereas the concentrations of fibrinogen, DD, FDP, PLG, TAT, PIC, t‑PAIC, and TM increased significantly. The divergence of these pregnancy-adapted intervals from standard non‑pregnant ranges is further illustrated in Fig. 4. This visualization underscores the critical importance of using trimester-specific, rather than general population, reference criteria for the accurate assessment of coagulation status during pregnancy.

**Table 2:** Median and 95% reference intervals for PT, APTT, TT, FBG, D-dimer, FDP, PLG, TAT, PIC, TM, and t-PAIC of pregnant women and non-pregnant women in trimester-specific groups

Parameter	Healthy non-pregnant women M (P2.5-P97.5)	First trimester M (P2.5-P97.5/< P95)	Second trimester M (P2.5-P97.5)	Third trimester M (P2.5-P97.5)
PT(S)	12.9(11.8 ~ 13.9)	13.1(12.1 ~ 14.4)	12.4(11.7 ~ 13.6)	12.3(11.4 ~ 13.3)
INR	1.01(0.89 ~ 1.12)	1.03(0.93 ~ 1.17)	0.96(0.89 ~ 1.09)	0.95(0.86 ~ 1.05)
APTT(S)	34.8(30.2 ~ 42.3)	34.4(29.5 ~ 40.8)	31.6(27.7 ~ 37)	31(27.8 ~ 35.8)
TT(S)	16.7(14.9 ~ 18.2)	16(14.7 ~ 17.5)	15.4(14.2 ~ 16.8)	15.5(14.4 ~ 16.8)
FBG(g/L)	2.62(1.93 ~ 3.80)	3.06(2.12 ~ 4.3)	3.97(3.04 ~ 5.56)	4.49(3.34 ~ 5.96)
DD(mg/l (FEU))	0.16(< 0.37)	0.25(< 0.84)	0.65(< 1.88)	1.13(< 2.48)*
FDP(mg/l)	1.8(< 3.46)	2(< 4.71)	3.1(< 7.02)	3.9(< 9.54)
PLG(%)	96(79 ~ 134)	100(80 ~ 126)	135(91 ~ 171)	140(89 ~ 187)
TM(TU/ml)	7.28(4.27 ~ 10.17)	7.14(4.71 ~ 10.87)	7.64(5.18 ~ 12.28)	8.1(5.69 ~ 11.47)
TAT(ng/ml )	1.76(< 4.01)	1.81(< 4.41)	5.76(< 10.16)	8.56(< 13.16)
PIC(µg/ml )	0.43(< 0.72)	0.51(< 0.89)	0.5(< 1.01)	0.45(< 1.09)
t-PAIC(µg/ml )	3.12(< 7.14)	2.65(< 6.24)	4.3(< 7.96)	5.81(< 10.47)

For PT, INR, APTT, TT, FBG, PLG, and TM, two-sided reference intervals were determined nonparametrically as the 2.5th to 97.5th percentiles. For DD, FDP, PIC, TAT, and t-PAIC, one-sided upper reference limits were set at the 95th percentile

* In the third-trimester pregnant cohort, a value of 0.5 mg/L FEU corresponds to the 4.3rd percentile

## Discussion

This study measured PT, INR, APTT, TT, FBG, D-dimer, FDP, PLG, TAT, TM, PIC, and t-PAIC levels in 253 non-pregnant healthy women and 1622 pregnant women. As shown in Supplementary Table 1, reference intervals for these parameters during pregnancy are not provided in the manufacturer’s instructions for use (IFU). Therefore, this study aimed to determine the normal ranges of these coagulation indices in women, especially during the hypercoagulable state of pregnancy, to address this gap. During the establishment of reference intervals, we strictly followed the requirements of the CLSI C28-A3 document to implement reasonable inclusion and exclusion criteria for the enrolled population. Considering the significant changes in coagulation function during pregnancy, participants were divided into three trimester groups, with each group meeting the sample size requirement of more than 120 cases.

Reference ranges for PLG throughout pregnancy have not been reported; only individual literature reports reference ranges in early pregnancy for small sample results [20], and there are no reliable literature reports on the trend of PLG during pregnancy for high-quality and large samples. In this study, plasma PLG levels were found to increase with advancing gestational weeks. The study showed that the reference interval (RI) of PLG in healthy non-pregnant women ranged from 79% to 134%. In early pregnancy, the RI of PLG ranged from 80% to 126%, in mid-pregnancy from 91% to 171%, and in late pregnancy from 89% to 187%. Other researchers can use this study as a reference interval.

The reference intervals for TAT, TM, PIC, and t-PAIC vary significantly across different research areas. For example, the research conducted by YANG et al. revealed no significant differences in TAT between the first and second trimesters in Changsha, China [16]. In contrast, the research conducted by Wu et al. and Xiao et al. found significant differences in TAT between the first and second trimesters in Beijing [21]and Shenzhen [17], China. It should be noted that the reference intervals for TAT vary considerably across these three regions. Similar difficulties faced by other projects suggest the need for formulating an individualized, local reference interval. However, when comparing intervals across studies, an important methodological constraint must be considered. A key limitation inherent in our study and many others in this field is the use of trimester-based groupings. As coagulation parameters change progressively, values at the end of one trimester (e.g., 13 weeks) are likely more similar to those at the start of the next trimester (e.g., 14 weeks) than to values at the beginning of its own trimester (e.g., 6 weeks). Therefore, the specific gestational week distribution of sampled women within each trimester in any given study can significantly influence the calculated reference interval. This methodological factor may contribute substantially to the apparent regional discrepancies reported in the literature, including the varying TAT intervals mentioned above. To achieve more precise and comparable benchmarks, future research should aim to establish reference intervals for narrower gestational windows or develop continuous reference curves across pregnancy. In the present study, TM levels slightly increased after the 21st week of pregnancy, similar to the study results of Wu et al. [21] and Xiao et al. [17] and slightly different from the study result of YANG et al. [16]. TAT and tPAI-C levels increased significantly with increasing gestational weeks, especially TAT, which also changed in late pregnancy and required more precise reference ranges. In contrast, The levels of PIC did not change significantly as the gestational weeks increased. However, by the 21st week of pregnancy, the dispersion of PIC was higher, with some subjects having PIC results two to three times higher than the median. This indicates that some pregnant women may be at an increased risk of bleeding after the 21st week of pregnancy.

The reference intervals for PT, INR, APTT, TT, FBG, D-dimer, and FDP levels were generally, but not entirely, consistent with those reported in other studies [6, 22]. Various studies have shown significant variation in formulating the reference intervals for D-dimer in pregnancy [6, 14, 22], mainly because D-dimer has not yet been standardized [23]. Furthermore, D-dimer detection systems are diverse, and the results of different detection systems have significant variations [24]. Therefore, establishing laboratory-specific reference intervals for D-dimer during pregnancy is critical. We discovered significant differences in D-dimer among each group. The upper reference limits (URLs, 95th percentile) for D-dimer, constituting the one-sided reference intervals, were determined as: <0.37 mg/L FEU for non-pregnant women, < 0.84 mg/L FEU for the first trimester, < 1.88 mg/L FEU for the second trimester, and < 2.48 mg/L FEU for the third trimester. The rise in D-dimer levels is primarily attributed to the activation of the coagulation system, which is accompanied by the activation of the fibrinolytic system.

With advancing gestational age, the synthesis of coagulation factors increases continuously. Consequently, the median values and lower reference limits of both APTT and PT decrease progressively, particularly in the third trimester. The upper reference limit of FBG reached 5.96 g/L, representing a 56.8% increase compared to the upper reference limit of 3.80 g/L in non-pregnant women. Concurrently, APTT and PT are shortened. A value of 0.5 mg/L FEU in the third-trimester pregnant cohort corresponds to the 4.3rd percentile, indicating that 95.7% of pregnant women in the third trimester have D-dimer levels exceeding the reference upper limit recommended by the kit. In the early stage of obstetric disseminated intravascular coagulation (DIC), the sufficient reserve of coagulation factors means that their consumption does not immediately cause a rapid prolongation of APTT or PT; in contrast, TAT can reflect the DIC state earlier, with its elevation preceding that of D-dimer. As DIC progresses further, D-dimer levels increase. As a direct marker of fibrinolytic activation, a significant elevation in PIC indicates an enhanced fibrinolytic state, which requires heightened vigilance for potential bleeding risks. In defining our healthy reference population, exclusions were based on clinical presentation and medical history, which excludes symptomatic individuals but cannot fully rule out asymptomatic hereditary or acquired thrombophilia, as systematic thrombophilia screening was beyond the study scope—a common limitation in similar reference interval studies, with future prospective studies including such screening needed to further validate the intervals; additionally, as a single-center, hospital-based study, our cohort may not fully represent China’s general pregnant population despite strict health criteria, introducing potential selection bias that requires multi-center or community-based studies for validation; the cross-sectional design provides only a population snapshot without individual longitudinal data, limiting understanding of physiological trajectories and necessitating prospective longitudinal studies to establish personalized trajectories and critical thresholds; finally, while the reference intervals were established in a healthy population and their potential clinical utility is discussed, the study was not designed to link parameter values to hard clinical outcomes (e.g., thrombosis, hemorrhage), requiring future prospective studies correlating laboratory values with clinical endpoints to define actionable decision points.

## Supplementary Information

Supplementary Material 1.

Supplementary Material 2.

## Data Availability

The data supporting this study’s findings are available on request from the corresponding author. The data are not publicly available due to privacy or ethical restrictions.

## Abbreviations

APTT: Activated partial thromboplastin time
PT: Prothrombin time
TT: Thrombin time
Fib: Fibrinogen
PLG: Plasminogen
TAT: Thrombin-antithrombin complex, TM, thrombomodulin, PIC, plasmin-α2 plasmin inhibitor complex, t-PAIC, tissue plasminogen activator/plasminogen activator inhibitor-I complex, GW, gestational week
t-PA: tissue plasminogen activator
FDP: Fibrin/fibrinogen degradation products
FEU: Fibrinogen equivalent units
RI: Reference interval
PAI: Plasminogen activator inhibitor
RI: Reference interval
