# Supplementary material for: Establishing trimester-specific reference intervals for coagulation parameters in pregnant women in China
Source: BMC Pregnancy Childbirth. 2026 May 18;26:736. doi: 10.1186/s12884-026-09296-7 (PMC13348816; doi:10.1186/s12884-026-09296-7)
Supplement: Supplementary file 1 — Supplementary Material 1. [file 12884_2026_9296_MOESM1_ESM.docx]

Supplementary Table 1. Reference intervals provided in the manufacturer’s instructions for use (IFU) for the general adult population.

| Parameter | Non-Pregnant Reference Interval (Manufacturer’s IFU) | Pregnancy-Specific Reference Interval (Manufacturer’s IFU) |  |
| --- | --- | --- | --- |
| PT(S) | Not provided | Not provided |  |
| INR | Not provided | Not provided |  |
| APTT(S) | Not provided | Not provided |  |
| TT(S) | <21 | Not provided |  |
| FBG(g/L) | 2–4 | Not provided |  |
| DD(mg/l (FEU)) | <0.5 | Not provided |  |
| FDP(mg/l) | <5 | Not provided |  |
| PLG(%) | 80–120 | Not provided |  |
| TM(TU/ml) | 3.82–13.35 | Not provided |  |
| TAT(ng/ml ) | <4.08 | Not provided |  |
| PIC(μg/ml ) | <0.85 | Not provided |  |
| t-PAIC(μg/ml ) | <10.52(Female) | Not provided |  |
