# Supplementary material for: Establishing trimester-specific reference intervals for coagulation parameters in pregnant women in China
Source: BMC Pregnancy Childbirth. 2026 May 18;26:736. doi: 10.1186/s12884-026-09296-7 (PMC13348816; doi:10.1186/s12884-026-09296-7)
Supplement: Supplementary file 2 — Supplementary Material 2. [file 12884_2026_9296_MOESM2_ESM.docx]

Supplementary Table 2. Intra-assay and inter-assay coefficients of variation for coagulation and fibrinolysis parameters.

| Parameter | intra- assay coefficients of variation | inter-assay coefficients of variation |  |
| --- | --- | --- | --- |
| PT(S) | 1.39% | 1.77% |  |
| INR | 1.78% | 2.20% |  |
| APTT(S) | 0.89% | 2.12% |  |
| TT(S) | 0.75% | 1.75% |  |
| FBG(g/L) | 2.32% | 2.72% |  |
| DD(mg/l (FEU)) | 3.30% | 7.14% |  |
| FDP(mg/l) | 3.74% | 9.05% |  |
| PLG(%) | 2.71% | 3.83% |  |
| TM(TU/ml) | 3.11% | 7.40% |  |
| TAT(ng/ml ) | 2.43% | 6.30% |  |
| PIC(μg/ml ) | 3.58% | 6.96% |  |
| t-PAIC(μg/ml ) | 1.48% | 9.03% |  |

Note: Intra-assay CV was derived from the instrument’s performance verification data, and inter-assay CV was obtained from the cumulative CV of internal quality control during the testing period.
